# Impact of antenatal care on neonatal mortality among neonates in Ethiopia: a systematic review and meta-analysis

**DOI:** 10.1186/s13690-020-00499-8

**Published:** 2020-11-10

**Authors:** Tadesse Tolossa, Ginenus Fekadu, Belayneh Mengist, Diriba Mulisa, Getahun Fetensa, Daniel Bekele

**Affiliations:** 1 Department of Public Health, Institute of Health Science, Wollega University, Nekemte, Ethiopia; 2 Department of Pharmacy, Institute of Health Science, Wollega University, P.O Box 395, Nekemte, Ethiopia; 3 Department of Public Health, College of Health Sciences, Debre Markos University, Debre Markos, Ethiopia; 4 School of Nursing and Midwifery, Institutes of Health Sciences, Wollega University, Nekemte, Ethiopia; 5 Department of Statistics, College of Natural Science, Dire Dawa University, Dire Dawa, Ethiopia

**Keywords:** Neonates, Neonatal mortality, Antenatal care, Ethiopia

## Abstract

**Background:**

As compared to other regions of the world, Sub Saharan Africa (SSA) is the region with the highest neonatal mortality and is the region showing the least progress in the reduction of newborn death. Despite better progress made in reducing neonatal mortality, Ethiopia contributes the highest rate of neonatal death in Africa. In Ethiopia, findings from few studies were inconsistent and there is a need to systematically pool existing data to determine the impact of antenatal care on neonatal mortality among mother-neonate pairs in Ethiopia.

**Methods:**

Published articles from various electronic databases such as Medline, Hinari, Pub Med, Cochrane library, the Web of Science, and Google Scholar were accessed. Also, unpublished studies from library catalogs were identified. All observational studies that were conducted on the association between antenatal care follow-up and neonatal mortality among neonates in Ethiopia were included. Data were extracted on the Microsoft Excel spreadsheet and analyzed using STATA 14.1 version. A random-effects model was used to estimate the pooled estimate with a 95% confidence interval (CI). Forest plots were used to visualize the presence of heterogeneity and estimate the pooled impact on antenatal care on neonatal mortality. The presence of publication bias was assessed by funnel plots and Egger’s statistical tests.

**Results:**

Initially, a total of 345 studies were accessed. Finally, 28 full-text studies were reviewed and fourteen studies fulfilled inclusion criteria and included in the final meta-analysis. The overall pooled estimate indicates the odds of neonatal death among neonates from women with antenatal care were 65% lower than those neonates from women who had no antenatal care follow-up (OR: 0.35, 95% CI: 0.24, 0.51).

**Conclusions:**

In this systematic review and meta-analysis, lack of ANC follow-up increase the probability of neonatal mortality as compared to having ANC follow-up. Thus, we will recommend for more coverages of appropriate antenatal care where risk groups can best be identified and managed.

**Supplementary Information:**

The online version contains supplementary material available at 10.1186/s13690-020-00499-8.

## Background

Globally in 2017, under-five mortality accounted 5.4 million deaths and an estimated 2.5 million deaths of under-five occur among newborns [1, 2]. Neonatal death contributes nearly 45% of under-five mortality, and the rate of death among newborn is higher than that of under-five death [3]. Worldwide in 2017, the neonatal mortality rate (NMRs) was 18 deaths per 1000 live births [4], with an estimated 1 million death happen on the first day and close to 1 million death within the first week of birth. Moreover, each year, around 1 million newborns develop long-term disability, including cerebral palsy and cognitive delays [2].

Neonatal period is the first 28 days of life, and is the time in which the child is most vulnerable to death, at a global rate of 19 deaths per 1000 live births. The NMR is an indicator of newborn care and directly reflects prenatal, intrapartum, and neonatal care [5]. The highest number of NMRs death occurred in Southern Asia and SSA with 39 and 38%, respectively [6]. From the top 10 countries contributing to NMRs, eight countries are from SSA and this region is the region with the highest child mortality rate in the world [2, 7]. In SSA, NMR ranges from 24 to 31 deaths per 1000 live births which is higher as compared to other regions of the world [2].

Reducing neonatal mortality is an essential part of the third Sustainable Development Goal (SDG) and is targeted to reduce the neonatal mortality rate to 12 or less per 1000 live births by 2030 [4]. Congruent to this, the government of Ethiopia aimed to end all preventable newborn diseases and developed a roadmap to drop under-five mortality rate to less than 20/1000 live births by 2035 [8]. In spite of all these plans and activities, neonatal mortality is still one of the great problems of this globe, with 18,000 under five and 7000 newborns dying every day, most of them from preventable causes [2].

Ethiopia is one of the top five countries contributes for half of neonatal mortality worldwide [6]. According to UNICEF 2018 report, NMRs in Ethiopia were 28 deaths per 1000 live births and are one of the top ten countries affected by NMR globally [2]. Mortality rates among under five children are a key output indicator for child health and survival, and, more broadly, for social and economic development. The neonatal mortality is a public health indicator that reflects the access of children and communities to basic health interventions such as vaccination, medical treatment of infectious diseases and adequate nutrition. The government of Ethiopia developed a National Newborn and Child Survival Strategy in 2014 which aimed to decrease NMR from 28/1000 live births in 2013 to 11/ 1000 live births in 2020 [8]. Even though the government of Ethiopia planned to decrease NMR by greater than half in 2020, still the NMR is at an alarming stage. According to mini EDHS 2019, the NMR in Ethiopia is 30 per 1000 live births [9] and preterm birth complications 26%, Intrapartum related events 30%, Sepsis/tetanus 18%, congenital abnormalities 11%, and pneumonia 8% are the leading causes for neonatal mortality [10]. In addition, factors such as lack of ANC follow-up [11, 12], Cesarian section [11], premature rupture of membrane [11, 13], Induced labor [13, 14], prolonged labor [14], lack of early initiation of EBF [14], being rural residence [13, 15, 16], poor wealth index [15, 16] and multiple birth [13, 16]. Despite lack ANC follow-up increase the occurrence of neonatal mortality, finding from developing countries presented inconclusive findings [17–30], which need meta-analysis Assessing the impact of ANC follow-up on neonatal mortality is important for addressing neonatal related problems and designing appropriate intervention which used to reduce neonatal mortality, particularly in resource-limited settings such as Ethiopia. Therefore, the objective of this systematic review and meta-analysis was to estimates the pooled impact of ANC on neonatal mortality among neonates in Ethiopia. The implications of the findings of our study are for national policymakers, program managers, and non-governmental organizations to reduce mortality among newborns in low-resource settings.

## Methods

### Search strategy

This systemic review and meta-analysis were conducted to assess the impact of antenatal care on neonatal mortality in Ethiopia. The existence of similar systematic reviews and meta-analysis which have been published on this topic was checked to prevent repetitions. Both published and unpublished studies were searched thoroughly using electronic databases such as Medline, Hinari, PubMed, Cochrane library, the Web of Science and Google Scholar using the key terms “ Antenatal care, Neonatal mortality, Neonatal outcomes, Obstetric care, Ethiopia”.

To find unpublished papers, some research centers, such as Addis Ababa University Digital Library and African digital library were searched. The search was conducted from November 1 to December 15, 2019. Pre-defined search terms were used to enable a comprehensive search strategy that included all the relevant studies. All fields within records and Medical Subject Headings (MeSH terms) were used to expand the search in advanced Pub Med search. The search strategy was prepared and modified for the various databases using important Boolean operators with initial keywords *(“Neonatal mortality” OR “neonatal death” OR “neonatal outcomes” AND “antenatal care” OR “obstetric care” AND “Ethiopia”).* The meta-analysis was reported using Preferred Reporting Items for Systematic Reviews and Meta-Analyses (PRISMA) guidelines.

### Selection and eligibility criteria

This systematic review included studies that were conducted on neonatal mortality and its association with antenatal care in Ethiopia. Study participants were all mother-neonates and cross sectional-studies, case-control, and cohort studies (both prospective and retrospective) which reported the association between ANC and neonatal mortality in Ethiopia was considered in this review. The included studies were written in the English language which was published in different journals and master’s thesis. We excluded articles that were found as abstract only since it was difficult to access all essential information required for the analysis. We tried to contact the primary authors of the articles with incomplete information and we excluded articles that were not accessible after contacting the principal investigator two times by email.

### Outcome measurement

The outcome of this systematic review and meta-analysis was the association between ANC follow-up and neonatal mortality. Neonatal mortality is death of the infant before 28 completed days. For the outcome variable, data were extracted in a format of two by two tables, and then the log OR was calculated based on the findings of the original studies. The systematic review and meta-analysis used the PICO (Population, Intervention, Comparison, and Outcomes) framework to assess the eligibility of the articles included. The study population (P) was all neonates in Ethiopia, the Intervention (I) was lack of ANC follow-up, the Comparison (C) was having greater than one ANC and the Outcomes (O) of the interest was neonatal death.

### Quality assessment and data extraction

The Joanna Briggs Institute Meta-Analysis of Statistics Assessment and Review Instrument (JBI-MAStARI) was used for critical appraisal. Initially, the reference management software (Endnote version X7) was used to combine database search results and to remove duplicate articles manually. Data were extracted by two data extractors using a standardized data extraction checklist. Then, studies excluded after a thorough assessment of their titles and abstracts. Full-text articles were evaluated for the remaining literature. Based on the pre-stated inclusion and exclusion criteria, eligibility of the studies was assessed. The checklist for data extraction contains the name of authors, publication year, region (the area where the study was conducted), study design, sample size, response rate and participants with the outcome (Table 1). Two reviewers (TT and BM) extracted the data using a standardized data extraction checklist on Microsoft excel. Discrepancies between two independent reviewers were reached on consensus by involving a third reviewer (DB).

**Table 1:** Summary of Included Studies regarding the impact of ANC follow-up on Neonatal mortality in Ethiopia, 2019

S.N	Primary author	Year	Study period	Region	Study design	Study Setting	sample size	ANC follow-up with neonatal mortality	Total ANC follow-up	Lack of ANC follow-up with neonatal mortality	Total number without ANC	OR (95% CI)
1	Yodit S.et al. [30]	1997	Nov - Dec, 1994	AA	Cross-sectional	Institution based	1365	70	1105	26	158	0.34 (0.21, 0.56)
2	Abdifatah E. et al. [23]	2018	25th of May 2017 to 10th of June, 2017.	Somali	Retrospective cohort	Institution based	792	37	703	8	90	0.57 (0.26, 1.26)
3	Gurmesa T.et al. [18]	2014	Sept 2012-Dec 2013	Oromia	Prospective follow-up	Community based	3463	78	2644	32	819	0.75 (0.49, 1.14)
4	Hirpha A. et al. [27]	2019	Jan 2015, to March 2016	Oromia	Case control	Institution based	300	32	135	68	165	0.44 (0.27, 0.73)
5	Negera W et al. [28]	2013	2011	EDHS data	Cross-sectional	Community based	8651	146	2895	371	5756	0.77 (0.63, 0.94)
6	Selamnesh T.et al. [29]	2019	Jan 2017 to June 2017	SNNP	Case control	Institution based	821	213	703	61	68	0.05 (0.02, 0.11)
7	Tufa K,. et al. [22]	2016	Jan to May 2015	Amhara	Case control	Community based	336	72	313	12	25	0.32 (0.14, 0.74)
8	Mihiretu A. et al. [20]	2017	July 1–30, 2015	SNNP	Cross-sectional	Institution based	300	10	152	42	149	0.18 (0.09, 0.37)
9	Elias M.et al. [26]	2018	March 2011- Dec 2012	Oromia	Case control	Community based	219	56	177	17	42	0.68 (0.34, 1.36)
10	Yared Asmare [24]	2018	March to April 1, 2018	AA	Retrospective cohort	Institution based	604	118	481	52	90	0.24 (0.15, 0.38)
11	Bogale Worku, Et al. [17]	2012	2001–2005	AA	Retrospective cohort	Institution based	3789	732	3288	106	311	0.55 (0.43, 0.71)
12	Elias M. et al. [25]	2019	2010–2014	Oromia	Retrospective cohort	Institution based	2090	107	1617	76	873	0.74 (0.55, 1.01)
13	Fillmon K. et al. [19]	2019	Feb 1, until Dec 30, 2013	Benishangul Gumuz	Case control	Community based	238	47	131	67	97	0.25 (0.14, 0.44)
14	Tujare T. et al. [21]	2019	2015 to 2017	SNNP	Retrospective cohort	Institution based	964	79	798	80	166	0.12 (0.08, 0.17)

*SNNP* Southern Nation Nationalities and People, *AA* Addis Ababa

### Statistical analysis and synthesis

Data were extracted in Microsoft Excel format and imported to STATA version 14 statistical software for analysis. The logarithm and standard error of the odds ratio (OR) for each included study were generated using the “generate” command. Cochran’s Q test (reported as *p*-value) and inverse variance index (I^2^) were used to check the presence of heterogeneity among the included studies. A high degree of heterogeneity was observed hence a random effect model was used for analysis to estimate the pooled impact of ANC on neonatal mortality. In addition, we conducted Meta-regression to identify the source of heterogeneity by using sample size and year of publication. A funnel plot of asymmetry was used to check the presence of publication bias. Furthermore, Egger and Begg’s statistical test was used to check the statistical significance of publication bias. Subgroup analyses by region (Addis Ababa (AA), Somali, Oromia, Ethiopia demographic health survey (EDHS), Southern Nations, Nationalities, and Peoples’ (SNNP), Amhara, Benishangul Gumuz) and study setting (community, facility based) of the included studies were carried out. The odds ratio of the association between ANC and neonatal mortality in the form of forest plot was reported.

## Results

### Search result

**Fig. 1:**
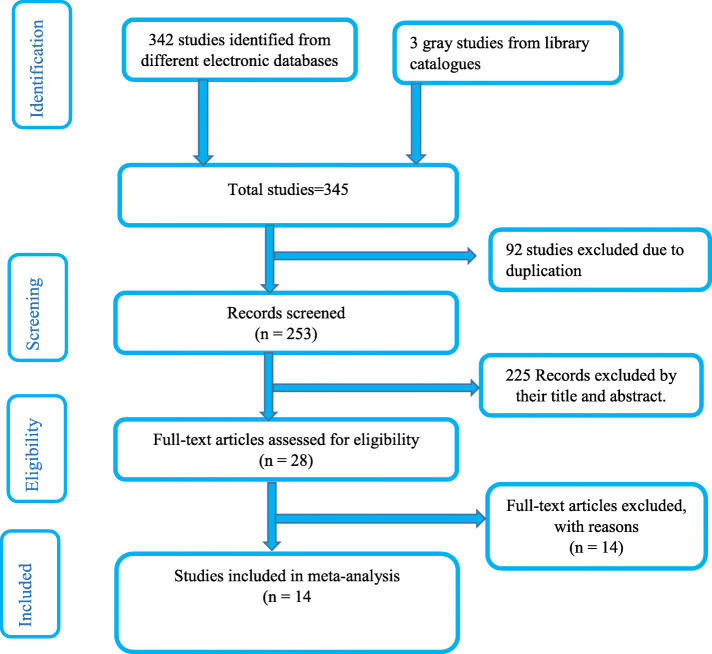
Flow diagram of the studies included in the meta-analysis

In the first step of our search, 345 studies were identified on neonatal mortality in Ethiopia through various electronic databases and library catalogs. Of these, 92 studies were excluded due to duplicates. From the remaining 253 studies, 225 articles were screened after reviewing their titles and abstracts based on the assessment as non-relevance to this study. The remaining 28 full-text articles were assessed for eligibility and 14 articles were excluded due to pre-determined eligibility criteria. Finally, 14 articles fulfilled the eligibility criteria and included in systematic review and meta-analysis (Fig. 1).

### Features of included studies

As shown below in Table 1, in the present meta-analysis 23,932 neonates were involved in the studies to estimates the pooled impact of antenatal care on neonatal mortality. All of the 14 articles included in this study were published from 1997 to 2019. Thirteen of the included studies were published in peer-reviewed journals and one study was an unpublished as a master’s thesis at Addis Ababa University [24]. Regarding study design, five of the studies are retrospective cohort study design [17, 18, 21, 24, 25], three are case-control [22, 26, 29], three cross-sectional study design [20, 28, 30] and one study is prospective cohort study design [23]. The sample size of the studies ranging from 219 to 8651. Of the fourteen studies, four studies were conducted in Oromia region [18, 25–27], Three from Addis Ababa [17, 24, 30], three from SNNP [20, 21, 29], One from Amhara region [22], One from Somali region [23], one from Benishangul region [19], and one study from EDHS data [28]. However, there were no studies reported from Gambella, Afar and Tigrai region (Table 1).

### Impact of ANC follow-up on neonatal mortality

The findings of single studies were inconsistent and inconclusive with the association between neonatal mortality and antenatal care which found to be significant in some studies and insignificant other. Of those studies that found a significant association between ANC and neonatal mortality, the strongest negative association was observed in the study conducted in the SNNP region, with an odds ratio of 0.05 (95% CI, 0.02, 0.11) [29].

**Fig. 2:**
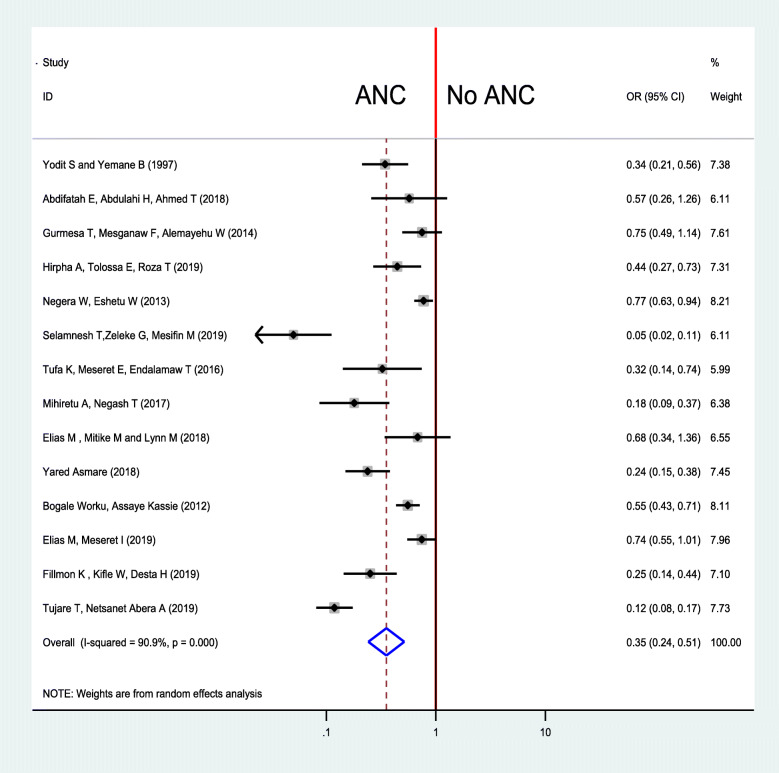
Forest plot for pooled impact of ANC follow up on neonatal among neonates in Ethiopia, 2019

**Fig. 3:**
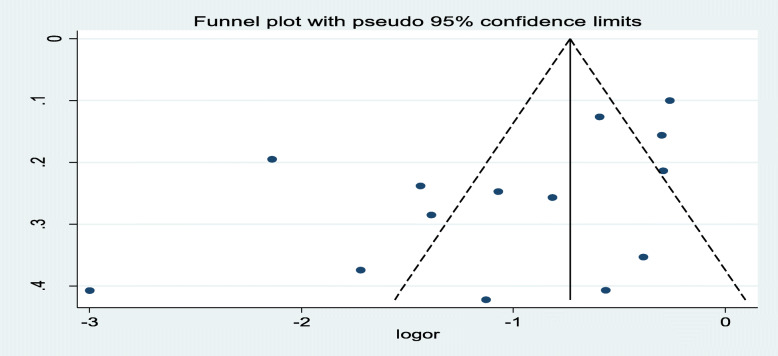
Funnel plot with 95% confidence limits of the pooled impact of ANC follow up on neonatal mortality in Ethiopia, 2019

In this study, the pooled odds ratio indicated that antenatal care was negatively associated with neonatal mortality (OR: 0.35, 95% CI: 0.24, 0.51). High heterogeneity (I^2^ = 90.9% and *p*-value < 0.001) was observed across the included studies; hence, a random-effects meta-analysis model was used to examine the association between ANC and neonatal care (Fig. 2). To identify the possible sources of heterogeneity, meta-regression was computed by busing sample size and year of publication but none of these variables were found to be statistically significant (Table 2). To see for the presence of publication bias, the graphical funnel plot (Fig. 3) and Egger’s test at 5% significance level were executed. The traditional funnel plot showed it to be asymmetric for publication bias. In addition, Egger’s test showed statistically significant for the presence of publication bias (*p* = 0.033).

**Table 2:** Related factors with the heterogeneity of the impact of ANC follow-up on neonatal mortality in Ethiopia, 2019

Variables	Coefficients	***p***-value
Publication Year	−0.0096438	0.796
Sample size	0.0001471	0.943

**Fig. 4:**
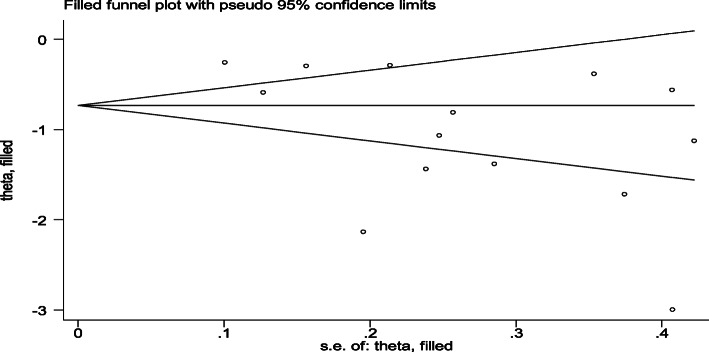
Result of trim and fill analysis for adjusting publication bias of the 14 studies, 2019

To reduce and adjust the publication bias in the studies, the trim and fill analysis was performed for estimation of the number of missing studies that might exist (Supplementary material 1). Trim and fill analysis is a nonparametric methods for estimating the number of missing studies that might exist and it helps in reducing and adjusting publication bias in meta-analysis. In trim and fill analysis, there was no studies imputed for missing studies and after adjustment for publication bias, the estimated pooled impact of ANC on neonatal mortality was the same with the main result of meta-analysis 0.35 (95% CI, 0.24, 0.51) (Fig. 4).

### Subgroup analysis

**Fig. 5:**
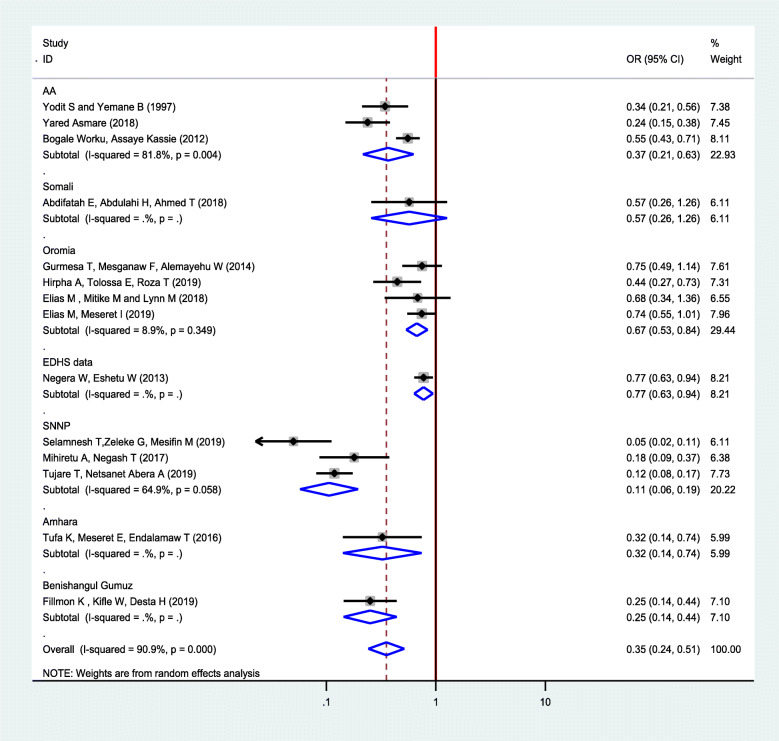
Sub group analysis for impact of ANC follow up on neonatal mortality based on the region where the studies were conducted in Ethiopia, 2019

We performed subgroup analysis based on the regions where the studies were conducted and study setting. Accordingly, subgroup analysis conducted by the region where the studies were conducted to reduce the possible random disparity between studies. The finding showed the strongest association between ANC and neonatal mortality was found in the study conducted in the SNNP region than other regions. The odds of neonatal mortality among women who had ANC follow-up was 89% lower compared to women who had no ANC follow-up in study conducted in the SNNP region (OR: 0.11, 95% CI: 0.06–0.19) (Fig. 5).

**Fig. 6:**
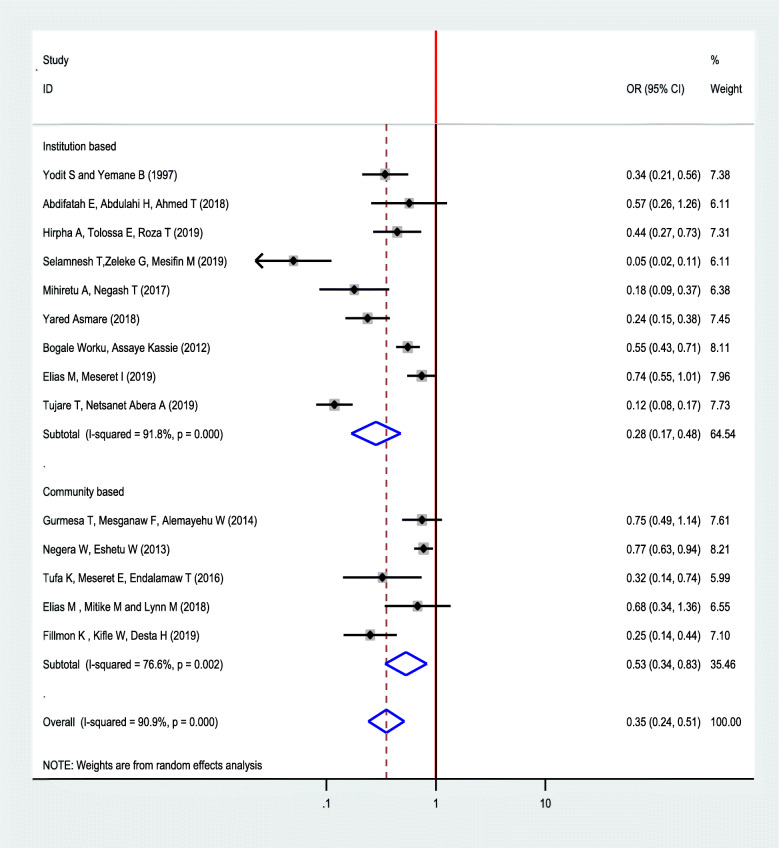
Sub group analysis for impact of ANC follow up on neonatal mortality based on the setting in Ethiopia, 2019

Moreover, subgroup analysis was conducted by study setting to minimize potential random variation between studies. Accordingly, the association between ANC and neonatal mortality were stronger in studies conducted at institutional setting than community-based study, in which the odds of neonatal mortality among women who had ANC follow-up was 72% lower compared to women who had no ANC follow-up in study conducted at institutions (OR: 0.28, 95% CI: 0.17, 0.48) (Fig. 6).

## Discussion

Obstetric care is a single most important determinant for the outcome of pregnancy of which antenatal care is recognized as an effective method of preventing adverse outcomes in mothers as well as their neonates. The current systematic review and meta-analysis, therefore, was conducted to assess the pooled association between antenatal care and neonatal mortality in Ethiopia. This systematic review and meta-analysis indicated the impact of ANC follow-up on neonatal mortality has a statistically significant negative association. Having ANC follow-up decrease the odds of neonatal mortality as compared to lack of ANC follow-up. Therefore, frequent and timely ANC follow-up is very important obstetric care to reduce neonatal mortality particularly, in low and middle-income countries.

The finding this systematic review and meta-analysis were similar to a study conducted in Kenya [31] where having ANC follow-up was found to have a significant (negative) effect on the likelihood of neonatal mortality. The finding is also in line with the study conducted in Sub-Saharan Africa countries [32] which indicated that prenatal care with skilled providers decreases the risk of neonatal mortality. Similarly, studies conducted in Nigeria [33], Zimbabwe [34] India [35] Afghanistan [36] and Brazil [37] also reported that ANC has a significant effect on the reduction of neonatal mortality. A previously conducted meta-analysis [38] reported a strong negative association of perinatal mortality with a lack of antenatal care.

This is because women who received ANC services which include a pregnancy checkup, provision health promotion, and disease prevention activities such as supplementation of iron/folic acid, tetanus toxoid vaccination, health education and counseling by a skilled health care provider. These services are crucial for the survival of the newborn and neonates as well.

Moreover, studies conducted in Bangladesh [39], Indonesia [40] and United Kingdom [41] revealed that the number of ANC visits determines perinatal and/ or outcomes which mean that as the number of visit increases, the probability of perinatal/ neonatal mortality decreases significantly. This is because as pregnant women come to health facilities frequently, they can access quality obstetric care in each visit. This is important to detect any deviation from normality so that problems are detected early and appropriate measures could be taken promptly.

The current review also performed subgroup analysis to identify the heterogenic characteristics of the included studies. A subgroup analysis was conducted by regions of the country. Accordingly, a higher association between ANC and neonatal mortality was observed among studies conducted in SNNP than in other regions. This could be because of social, economic, and cultural differences between regions where studies were conducted. A subgroup analysis by study setting indicated a strong association was observed in institutional studies than community-based studies which are probably the difference in the characteristics of study participants of the included studies.

This study has its own implication for future researchers. It guides researchers to conduct large scale and follow-up study from primary data to see the relationship between ANC follow-up and neonatal mortality by including other potential factors which contribute for neonatal mortality.

### Strengths and limitations of the study

This review has several strengths including; this review focus on the most important determinant of neonatal mortality which is ANC, essential care during pregnancy. Moreover, we used comprehensive search strategies and PRISMA checklist to improve the quality of the review. Whereas, this review has limitations such as the review included studies that were published only in the English language. Also, the other limitation of this review is that the protocol of this manuscript was not registered prospectively.

## Conclusions

In this systematic review and meta-analysis, the absence of antenatal care booking positive impact on neonatal mortality which might be due to a lack of prevention of most other preventable factors during pregnancy. Intervention that focus on educating mothers on the importance of antenatal visits, as well as ensuring screening, detection, monitoring, and management of maternal conditions during the pregnancy, could help to reduce NMR. Provision of the continuum of care from pregnancy through delivery to the post neonatal period should be provided so as to decrease neonatal mortality. In addition, since the finding is heterogeneous across the regions of the country, culture- and context-specific maternal health education needs to be encouraged.

## Supplementary Information

**Additional file 1.**

## Data Availability

The datasets analyzed during the current study are available from the corresponding author upon reasonable request.

## Abbreviations

ANC: Antenatal care
CI: Confidence Interval
SSA: Sub Saharan Africa
SDG: Sustainable Development Goals
OR: Odd Ratio
SNNP: Southern Nation Nationalities and People
WHO: World Health Organization
