# Supplementary material for: Impact of antenatal care on neonatal mortality among neonates in Ethiopia: a systematic review and meta-analysis
Source: Arch Public Health. 2020 Nov 10;78:114. doi: 10.1186/s13690-020-00499-8 (PMC7653817; doi:10.1186/s13690-020-00499-8)
Supplement: Supplementary file 1 — Additional file 1. [file 13690_2020_499_MOESM1_ESM.docx]

**Supplementry material 1: Result of trim and fill analysis**
